# Cytoreductive surgery followed by chemotherapy versus chemotherapy alone for recurrent platinum-sensitive epithelial ovarian cancer (SOCceR trial): a multicenter randomised controlled study

**DOI:** 10.1186/1471-2407-14-22

**Published:** 2014-01-14

**Authors:** Rafli van de Laar, Petra LM Zusterzeel, Toon Van Gorp, Marrije R Buist, Willemien J van Driel, Katja N Gaarenstroom, Henriette JG Arts, Johannes CM van Huisseling, Ralph HM Hermans, Johanna MA Pijnenborg, Eltjo MJ Schutter, Harold MP Pelikan, Jos HA Vollebergh, Mirjam JA Engelen, Joanna IntHout, Roy FPM Kruitwagen, Leon FAG Massuger

**Affiliations:** 1 Department of Obstetrics and Gynecology, Radboud University Medical Centre, PO Box 9101, 6500, HB, Nijmegen, The Netherlands; 2 Department of Obstetrics and Gynaecology, Maastricht University Medical Center, GROW -School for Oncology and Developmental Biology, Maastricht, The Netherlands; 3 Department of Obstetrics and Gynaecology, Academic Medical Center, Amsterdam, The Netherlands; 4 Department of Obstetrics and Gynaecology, Netherlands Cancer Institute/Antoni van Leeuwenhoek Hospital, Amsterdam, The Netherlands; 5 Department of Obstetrics and Gynaecology, Leiden University Medical Center, Leiden, The Netherlands; 6 Department of Gynaecology, University Medical Center Groningen, Groningen, The Netherlands; 7 Department of Obstetrics and Gynaecology, Groene Hart Hospital, Gouda, The Netherlands; 8 Department of Obstetrics and Gynaecology, Catharina Hospital, Eindhoven, The Netherlands; 9 Department of Obstetrics and Gynaecology, TweeSteden Hospital, Tilburg, The Netherlands; 10 Department of Obstetrics and Gynaecology, Medical Spectrum Twente, Enschede, The Netherlands; 11 Department of Obstetrics and Gynaecology, Bronovo Hospital, Den Haag, The Netherlands; 12 Department of Obstetrics and Gynaecology, Bernhoven Hospital, Uden, The Netherlands; 13 Department of Obstetrics and Gynaecology, Atrium Medical Center, Heerlen, The Netherlands; 14 Department for Health Evidence, Radboud University Medical Center, Nijmegen, The Netherlands

**Keywords:** Secondary cytoreductive surgery, Recurrent, Platinum-sensitive, Ovarian cancer

## Abstract

**Background:**

Improvement in treatment for patients with recurrent ovarian cancer is needed. Standard therapy in patients with platinum-sensitive recurrent ovarian cancer consists of platinum-based chemotherapy. Median overall survival is reported between 18 and 35 months. Currently, the role of surgery in recurrent ovarian cancer is not clear. In selective patients a survival benefit up to 62 months is reported for patients undergoing complete secondary cytoreductive surgery. Whether cytoreductive surgery in recurrent platinum-sensitive ovarian cancer is beneficial remains questionable due to the lack of level I-II evidence.

**Methods/Design:**

Multicentre randomized controlled trial, including all nine gynecologic oncologic centres in the Netherlands and their affiliated hospitals. Eligible patients are women, with first recurrence of FIGO stage Ic-IV platinum-sensitive epithelial ovarian cancer, primary peritoneal cancer or fallopian tube cancer, who meet the inclusion criteria. Participants are randomized between the standard treatment consisting of at least six cycles of intravenous platinum based chemotherapy and the experimental treatment which consists of secondary cytoreductive surgery followed by at least six cycles of intravenous platinum based chemotherapy. Primary outcome measure is progression free survival. In total 230 patients will be randomized. Data will be analysed according to intention to treat.

**Discussion:**

Where the role of cytoreductive surgery is widely accepted in the initial treatment of ovarian cancer, its value in recurrent platinum-sensitive epithelial ovarian cancer has not been established so far. A better understanding of the benefits and patients selection criteria for secondary cytoreductive surgery has to be obtained. Therefore the 4^th^ ovarian cancer consensus conference in 2010 stated that randomized controlled phase 3 trials evaluating the role of surgery in platinum-sensitive recurrent epithelial ovarian cancer are urgently needed. We present a recently started multicentre randomized controlled trial that will investigate the role of secondary cytoreductive surgery followed by chemotherapy will improve progression free survival in selected patients with first recurrence of platinum-sensitive epithelial ovarian cancer.

**Trial registration:**

Netherlands Trial Register number: NTR3337.

## Background

Since overall survival is poor for patients with recurrent platinum-sensitive ovarian cancer, improvement of treatment is needed for these patients. Standard therapy in patients with platinum-sensitive recurrent ovarian cancer is platinum-based chemotherapy [1]. In two large prospective trials this resulted in a median overall survival of 18 and 29 months [2,3]. The recently published OCEANS trial, compared carboplatin in combination with gemcitabine+/- bevacizumab, and reported a median overall survival of 33-35 months [4]. The role of secondary debulking surgery in recurrent ovarian cancer is not clear, and current practice differs widely between institutions. Several studies, nearly all retrospective, with highly selected patients, have demonstrated a survival benefit with survival rates up to 62 months for patients undergoing complete secondary cytoreductive surgery [5]. Three studies evaluated the predictive factors to accomplish complete secondary cytoreductive surgery. The first, the Arbeitsgemeinschaft Gynäkologische Onkologie (AGO) DESKTOP I study, found three predictive factors for complete resection: good performance status, no or small volume of ascites ( <500 ml), and complete primary surgery or FIGO I/II. In these selected patients (AGO-score positive), complete resection was achieved in 79% [6]. The second, the DESKTOP II trial, prospectively validated the AGO score and confirmed a complete resection rate of 76% [7]. However in this validation study only 57% of the score-positive patients underwent a secondary debulking which may have resulted in selection-bias and subsequently reduced validation. The third study of Tian et al. developed a risk model with six predictive factors (FIGO stage, residual tumor after primary surgery, PFI (months), ECOG performance status, CA125 at recurrence (U/ml) and ascites at recurrence) [8]. This model categorizes patients as low or high risk. In the low-risk group the proportion of complete cytoreduction was 53.4% compared with 20.1% in the high-risk group. Inclusion criteria of the current study are based on predictive factors as observed in the mentioned studies. Due to the retrospective nature of most studies reporting on secondary cytoreductive surgery, information on surgery related morbidity and second line chemotherapy is scarce. If secondary cytoreductive surgery causes chemotherapy delays and/or dose reductions, is therefore unknown Bristow et al. reported peri-operative morbidity with a weighted mean of 19.2% [5] which seems comparable to morbidity in primary cytoreductive surgery [9]. Whether cytoreductive surgery in recurrent platinum-sensitive ovarian cancer is really beneficial remains questionable due to the lack of level I-II evidence [10]. Therefore, the 4^th^ ovarian cancer consensus conference in 2010 stated that randomized controlled phase 3 trials that evaluate the role of surgery in platinum-sensitive recurrent ovarian cancer should be considered as a priority [11].

### Objective

The aim of this study is to determine whether secondary cytoreductive surgery followed by platinum-based chemotherapy increases progression-free survival in patients with recurrent platinum sensitive epithelial ovarian cancer, primary peritoneal cancer or fallopian tube cancer.

#### *Primary outcome measure*

Primary outcome measure is progression free survival, defined as the interval between the date of randomisation and the date of progressive disease or death of any cause, whatever occurs first. Progressive disease defined as clinical and radiological signs of recurrence (RECIST 1.1 criteria) [12] or elevated CA 125 (GCIG criteria) and radiological signs of recurrence (RECIST 1.1 criteria) [13].

#### *Secondary outcome measures*

Secondary outcome measures are overall survival, progression free survival since start therapy and after the sixth chemotherapy cycle, quality of life during two years after treatment (EORTC QLQ-C30 version 3.0 and QLQ-OV28, EQ-5D), surgery related morbidity and mortality, toxicity (NCI-CTC Toxicity Scale Version 4.0), tumor response following treatment (RECIST and GCIG criteria).

## Methods/Design

### Study design

**Figure 1:**
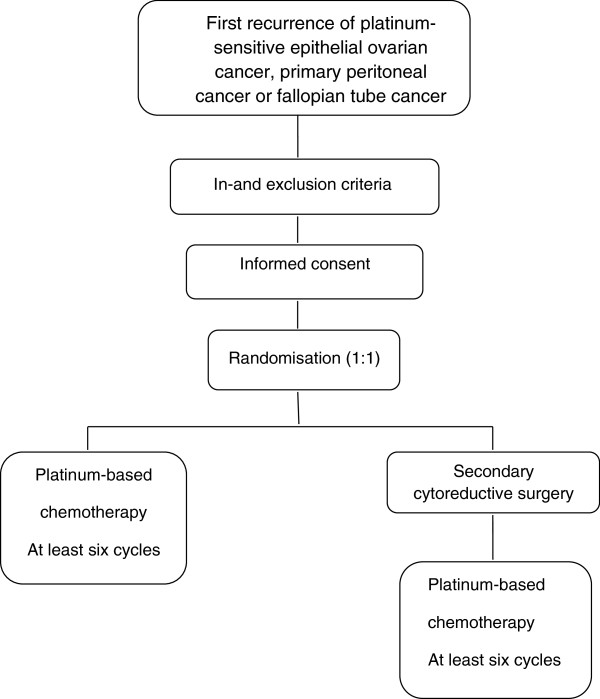
SOCceR trial study design.

This study is a multicenter prospective randomised controlled phase III trial in which all nine Dutch gynaecological oncology centres and affiliated hospitals are participating. Patients with first recurrence of platinum-sensitive epithelial ovarian cancer, primary peritoneal cancer or fallopian tube cancer will be randomly assigned to the control arm and experimental arm in a 1:1 ratio. In the control arm patients receive the standard treatment for platinum-sensitive recurrent ovarian cancer of at least six cycles of intravenous platinum containing chemotherapy. In the experimental arm treatment consists of secondary cytoreductive surgery followed by at least six cycles of intravenous platinum containing chemotherapy (Figure 1).

### Eligibility criteria

#### *Inclusion criteria patients*

Patients, ≥ 18 years, with first recurrence of platinum-sensitive (≥ 6 months after completion of front-line platinum-taxol chemotherapy) epithelial ovarian cancer, primary peritoneal cancer or fallopian tube cancer, are eligible for this study and can be randomised after given informed consent. First recurrence defined as clinical and radiological signs of recurrence (RECIST 1.1 criteria) or elevated CA 125 (GCIG criteria) and radiological signs of recurrence (RECIST 1.1 criteria).

Inclusion criteria are FIGO stage Ic-IV (FIGO system 1988), first-line treatment consisted of complete or optimal (≤ 1 cm) cytoreductive surgery and (neo-adjuvant) platinum-taxol based chemotherapy, ascites < 500 ml (pocket < 8 cm on ultrasound examination), complete resection seems possible (estimated by a gynaecologic oncologist), good performance status (ECOG 0-1) and administration of platinum based chemotherapy is possible (appropriate laboratory values).

#### *Exclusion criteria patients*

Exclusion criteria are non-epithelial or borderline ovarian tumours, platinum-refractory or resistant tumour, secondary or later recurrence, prior or already planned therapy with respect to recurrence, any disease, medical history or medication not allowing surgery and/or platinum based chemotherapy, concurrent treatment for other primary malignancy except for carcinoma in situ and basal or squamous cell carcinoma of the skin or participation in interfering trial.

### Requirement for participating hospitals

All hospitals can participate in this trial on the condition that chemotherapy in both arms is given by a registered medical oncologist and secondary cytoreductive surgery is performed by a gynaecological oncologist in one of the nine gynaecological oncology centres or one of their affiliated hospitals certified as an ovarian cancer treatment center. In addition, approval by the local Institutional Medical Ethics Committee (MEC) must be settled.

### Patient recruitment

All eligible patients will be evaluated by a multidisciplinary team consisting of at least a gynaecological oncologist, a medical oncologist and an experienced radiologist. Confirmation of recurrent ovarian cancer by cytological or histological investigation is warranted if there is any doubt regarding an abnormal finding on CT-scan. Diagnostic laparoscopy is allowed to estimate whether complete resection is possible and has to be performed by a gynaecological oncologist. All patients who meet the inclusion criteria, will be asked to participate in this study. After obtaining written informed consent, randomisation can take place.

### Randomisation

Randomisation is performed by accessing a central internet-based randomisation programme. Stratification will be done for gynaecologic-oncologic center hospital and completeness of primary cytoreductive surgery (complete vs optimal). Patients will be randomly allocated to both arms in a 1:1 ratio. To ensure patients privacy, patients will be registered by study trial number after randomisation.

### Pre-treatment evaluation

Patients in both treatment arms should be asked to fill out a baseline quality of life questionnaire (EORTC QLQ-C30 version 3.0 and QLQ-OV28, EQ-5D). Furthermore, CA 125 must be determined and a Case Report Form (CRF)with respect to baseline characteristics must be completed.

### Interventions

#### *Control arm*

Patients in the control arm will receive at least six courses of platinum-containing chemotherapy. The first course should be given as soon as possible but within 4 weeks after randomisation. Chemotherapy dose and schedule, combination with other chemotherapeutic agents and pre-and post-chemotherapy medication and hydration is left to the discretion of the participating center. Before every chemotherapy cycle, hematological, renal, and hepatic function will be determined and toxicity is scored using the common toxicity criteria for adverse effects (CTCAE) version 4.0.

#### *Experimental arm*

Secondary cytoreductive surgery must be performed within 4 weeks after randomisation. All patients will be prepared for the possibility of a bowel stoma. Surgery is carried out under antibiotic prophylaxis. The combination of antibiotics will be according local standards. The objective of the surgical intervention is complete resection of all visible disease. The extensiveness of surgery is left to the discretion of the gynaecological oncologist. The surgical procedure should start with obtaining a peritoneal fluid sample for cytology. Patients who have at least 500 ml of peritoneal fluid at the time of laparotomy are considered positive for ascites. To gain proper insight in the tumor spread, all adhesions from any previous surgery have to be excised. This is followed by a thorough inspection and palpation of the intra-abdominal and retroperitoneal contents. The extend of tumor growth is carefully recorded in eight abdominal regions. In case of peritonitis the number of tumor deposits will be recorded. Based on this information, a plan for dissection is made. At the end of the cytoreductive procedure, the extensiveness of surgery, duration of the operation, the amount of blood loss and the amount of residual tumor is recorded. Secondary cytoreductive surgery is considered complete if there is no tumor left, optimal if the largest residual tumor is ≤ 1 cm and suboptimal if more than 1 cm residual tumor (diameter (length, width or depth) of the separate tumor depositions) is left. The first chemotherapy course should be given as soon as possible but within 6 weeks after surgery.

### Post-treatment evaluation

In both treatment arms, evaluation of response will take place before the fourth chemotherapy course and within six weeks after the sixth chemotherapy course. This evaluation consists of determining CA 125 (GCIG criteria) and performing a CT-scan (RECIST 1.1). Toxicity is scored using the common toxicity criteria for adverse effects (CTCAE) version 4.0. Patients will be asked to fill out a quality of life questionnaire (EORTC QLQ-C30 version 3.0 and QLQ-OV28, EQ-5D). After cytoreductive surgery in the experimental arm, haemoglobin must be determined. A Case Report Form (CRF) with respect to the chemotherapeutic treatment and operation must be completed.

### Follow-up

As the primary outcome measure of this study is progression free survival, strict follow-up is warranted. After completion of therapy, the patients will be evaluated by their gynaecological oncologist/gynaecologist and medical oncologist every three months for the first two years and twice annually during the third, fourth and fifth year. At each outpatient control, patient’s history need to be obtained and CA 125 will be determined. A CT-scan has to be performed if there are clinical symptoms and CA 125 is in the normal range (0-35 U/ml) or if CA 125 is increased according to the GCIG criteria and there are no clinical symptoms. As overall survival is one of the secondary endpoints, follow-up of each patient in the study will be at least three years with a maximum of five years. Any treatment for second, third or later recurrence has to be documented. All data will be registered on a follow-up Case Record Form (CRF).

#### *Quality of life*

Health-related quality of life (HRQL) is one of the secondary endpoints in this study. It will be assessed with self-reported questionnaires (EORTC QLQ-C30 version 3.0 and QLQ-OV28, EQ-5D) to evaluate the impact of secondary cytoreductive surgery added to platinum based chemotherapy. The questionnaires have to be filled out after the sixth chemotherapy cycle and at 3, 6, 9, 12, 18 and 24 months following treatment.

#### *Assessment of serious adverse events*

A serious adverse event (SAE) related to surgery and/or chemotherapy will be collected and recorded on the Serious Adverse Event Report Form during every trial phase. They all must be reported to the principal investigator within 24 hours and will be followed until they have abated or until a stable situation has been reached.

### Statistical analysis

#### *Sample size*

Progression free survival of patients in the control arm is estimated at ten months. An increase in progression free survival of at least five months in favor of the experimental treatment is considered acceptable given the expected surgery related morbidity. To detect a constant hazard ratio of 1.5 with a 0.05 two-sided significance level and a power of 80%, 104 patients should be enrolled in each arm. Assuming an accrual period of five years, three years of follow-up and 10% loss to follow up, 230 patients need to be included.

### Interim analysis

An interim analysis for efficacy is planned at two months after randomisation of the last patient. Whatever the outcome of the interim analysis, all randomised patients will be followed until 36 months after randomisation of the last patient. This means that even in case of a statistically significant treatment effect, the trial will not be stopped. The objective of this interim analysis is that patients who are not participating in this study might profit in case of early positive results. The Peto-Haybittle rule, that requires P <0.001 as evidence, will be applied. This leaves the significance level of the final primary efficacy analysis at 0.0498.

### Data analysis

The results of the study will be analyzed according to the intention to treat principle. All patients will be included in the analysis and patients lost to follow-up will be regarded as censored observations. Progression free and overall survival in both treatment arms will be calculated by the Kaplan-Meier method and compared by using the log-rank test, assuming that the hazard rates in the two treatment arms are roughly proportional over time. If mortality due to surgery is unexpectedly high, resulting in proportional hazard (PH) assumptions that are not valid, the primary comparison of the treatment groups will be based on the one-year progression-free disease rates. In addition, progression free and overall survival since start therapy and after the sixth chemotherapy cycle will be compared.

To adjust for baseline covariates, we will also estimate the treatment effect by Cox’s proportional hazard (PH) regression model, given that the PH assumptions are valid. If the mortality due to surgery is unexpectedly high, resulting in PH assumptions that are not valid, progression free survival since start chemotherapy will be analyzed. At least the following baseline covariates will be considered: completeness of primary surgery, FIGO stage, ECOG performance status, age, disease free interval and CA-125 at recurrence. The significance level of the final primary analysis will be set at 0.0498, and for the interim analysis at 0.001. For all other analyses, 0.05 will be used in the report of study results.

Treatment related morbidity at 30 days after surgery will be analysed with descriptive statistics. Logistic regression will be used to study relations between clinical variables and morbidity. Toxicity after the third and sixth chemotherapy course will be evaluated by means of descriptive statistics and by using an unpaired Student’s t-test or the two-sided Mann-Whitney test for the continuous variables. Comparisons of the proportions of toxicity between the 2 arms will be done by use of a two-sided chi-square test or a two-sided Fisher’s exact test if the number of expected patients in a given category is less than five.

Quality of life scores will be summarized per time-point, for those subjects, who are alive, once for the original values and once for the changes from baseline. Only subjects who have filled in a baseline questionnaire will be included in the analysis. In addition to descriptive statistics, the Quality Adjusted Life Years (QALY) will be analysed. QALYs will be based on the EQ-5D-3 L questionnaire. The repeated measures indexes will be combined over time using the trapezium rule (area under the Quality Adjusted Life Year curve).

### Safety reviews and monitoring

The SOCceR study has established an Independent Data Safety Monitoring Board (DSMB) comprising of independent experts who have no conflict of interest and agree with the outline of the protocol. The committee will meet once a year. Following this meeting, the DSMB will report to the Study Coordinators about (serious) adverse events, whether or not recruitment is on target and the compliance with the QoL assessments is adequate. The committee may recommend changes in the conduct of the trial and exclusion of a single center if excessive rates of morbidity are present. All data presented at this meeting will be considered confidential. During the study, the committee may decide to change the frequency of discussion.

Safety reviews are planned primarily to guard against unfavorable results in the experimental arm. Death and failure rates and SAE reports for both treatment arms will be closely monitored in order to pick up any (unexpected) trends. Safety reviews will be presented confidentially to the DSMB every six months, and/or at request of the DSMB. These biannual reviews will include data on number and causality of deaths, number of treatment failures and serious adverse events. The DSMB can recommend to modify or stop the study prematurely, if number and causality of deaths, number of treatment failures and serious adverse events are significantly greater than was foreseen in the literature. The assessment of the DSMB, will be presented to the principal investigators and will be reported in the annual progress report to the accredited Medical Ethics Committee.

### Ethics

The study will be conducted in full conformance with the ethical principles of the Declaration of Helsinki and in accordance with the Medical Research Involving Human Subjects Act (WMO). It has been approved by the Radboud Medical-Ethical Committee (ref. no MEC 2011/426). The protocol is registered in the Dutch Trial Register number NTR333. Before the start in other centres, the protocol will be approved by local medical ethical committees. Ethical approval has already been obtained by the following local Ethics committees: Clinical Trial Center of the Maastricht University Medical Center, Ethics committee of the Academic.

Medical Centre Amsterdam, Ethics committee of the Netherlands Cancer Institute/Antoni van Leeuwenhoek Hospital, Committee of medical Ethics of the Leiden University Medical Center, Ethics committee of the University Medical Center Groningen, Landsteiner Institute of the Groene Hart Hospital, Ethics committee of the Catharina Hospital, Science agency of the TweeSteden Hospital, Ethics committee of the Medical Spectrum Twente, Ethics committee of the Bronovo Hospital, Ethics committee of the Bernhoven Hospital and Ethics committee of the Atrium Medical Center.

The rationale, design and aims of the study will be explained to each patient along with the specific information on the respective treatment arms. The principles of randomisation and registration and the follow-up procedure will be clarified. The patient will receive written patient information and will have ample opportunity to ask questions. The patient will have sufficient time to consider the study before deciding to participate. Written informed consent of the patient is required before randomisation. Patients will also receive written information on the trial insurance that covers for damage through injury or death caused by the study.

### Quality assurance

To enable peer review and/or inspections from Health Authorities, the principal investigator will keep records, including the identity of all participating subjects (sufficient information to link records, e.g. CRFs and hospital records), all original signed Informed Consent Forms and copies of all CRFs, for 15 years.

## Discussion

### Other clinical trials

Currently, there are two other ongoing randomized controlled trials: the DESKTOP III trial (NCT01166737) and the GOG 213 trial (NCT00565851). The DESKTOP III trial will compare overall survival in patients with platinum-sensitive recurrent ovarian cancer with a positive AGO-score randomized to cytoreductive surgery followed by chemotherapy of physician’s choice versus chemotherapy of physician’s choice alone whereas the GOG 213 trial will determine if surgical secondary cytoreduction in addition to adjuvant chemotherapy comprising carboplatin and paclitaxel with or without bevacizumab increases the duration of overall survival of patients with recurrent platinum-sensitive ovarian epithelial cancer, primary peritoneal cavity cancer, or fallopian tube cancer. A better understanding of the real advantages and disadvantages and patient’s selection criteria for secondary cytoreductive surgery will be achieved after the completion of these three ongoing trials.

## Competing interests

The authors declare that they have no competing interests.

## Authors’ contributions

LFAGM, RFPMK, RL, PLMZ, TVG, JI were involved in conception and design of the study. RL drafted the first manuscript. All authors edited the manuscript and read and approved the final draft.

## Pre-publication history

The pre-publication history for this paper can be accessed here:

http://www.biomedcentral.com/1471-2407/14/22/prepub
